# Assessing Multiple Agronomic Functions of a Winter Pea (*Pisum sativum* L.) Variety Across Different Uses

**DOI:** 10.3390/plants15081226

**Published:** 2026-04-16

**Authors:** Ana Uhlarik, Bojan Vojnov, Marjana Vasiljević, Svetlana Vujić, Djordje Krstić, Željko Dolijanović, Srđan Šeremešić

**Affiliations:** 1 Institute of Field and Vegetable Crops, National Institute of the Republic of Serbia, Maksima Gorkog 30, 21000 Novi Sad, Serbia; ana.uhlarik@ifvcns.ns.ac.rs (A.U.); marjana.vasiljevic@ifvcns.ns.ac.rs (M.V.); 2 Faculty of Agriculture, University of Novi Sad, Sq Dositeja Obradovića 8, 21000 Novi Sad, Serbia; antanasovic.svetlana@polj.uns.ac.rs (S.V.); djordje.krstic@polj.uns.ac.rs (D.K.); srdjan.seremesic@polj.uns.ac.rs (S.Š.); 3 Faculty of Agriculture, University of Belgrade, Nemanjina 6, 11080 Belgrade, Serbia; dolijan@agrif.bg.ac.rs

**Keywords:** pea, protein composition, cover crop, biomass, green manure, soil nitrate, multifunctionality, sustainable agriculture

## Abstract

Pea (*Pisum sativum* L.) is a multifunctional legume of growing importance in sustainable cropping systems. This study presents an integrative assessment of a forage pea variety across multiple agronomic functions under temperate continental conditions. Results from three environmentally comparable field trials were synthesized to evaluate (i) grain yield and protein traits, (ii) biomass production and nutrient accumulation in cover cropping systems, and (iii) effects on soil nitrate dynamics and maize (*Zea mays* L.) yield. Compared with vegetable- and dry-seed-type genotypes, the forage-type cultivar exhibited greater plant height and lodging tendency, moderate grain yield, and elevated protein content (28.8%), characterized by a legumin-dominated protein profile. As a winter cover crop grown in mixture with oat (*Avena sativa* L.), pea produced lower total biomass than rye (*Secale cereale* L.) but showed substantially higher nitrogen concentrations (2.93–3.01%), indicating enhanced nitrogen input potential. In crop rotation, pea-based treatments significantly affected soil nitrate distribution and maize productivity. Complementary resource use in pea-based systems enhanced biomass production, supporting forage and green manure functions while contributing to soil fertility and system stability. Its morphological and physiological adaptability enables integration into diverse production models, from intensive to regenerative systems. Overall, pea should be regarded not merely as a single crop, but as a strategic component of diversified farming systems aimed at increasing protein yield, optimizing inputs, improving soil quality, and strengthening the long-term sustainability of agroecosystems.

## 1. Introduction

Pea *(Pisum sativum* L.) is one of the most widely cultivated species within the family Vicieae (*Fabaceae*) and represents an important legume grown worldwide [1], which can be sown as a spring or winter crop, depending on the climate. Due to its broad adaptability, pea is used for diverse purposes, including dry grain production, vegetable consumption, forage, and as a component of sustainable cropping systems [2,3]. Accordingly, pea is commonly classified into forage, vegetable, and dry-seed types, reflecting the multifunctional role of this species in modern agriculture. In recent years, pea has gained increasing attention as a strategic crop for sustainable food and feed production, owing to its ability to provide high-quality plant protein while simultaneously contributing to soil fertility and crop rotation diversity [4,5,6]. Pea seeds are characterized by a relatively high crude protein content, typically ranging from 13.7% to 33%, depending on genotype and growing conditions [7,8,9,10,11]. Numerous studies have demonstrated that both genetic background and environmental factors significantly influence protein content and composition in pea seeds [7,12,13,14]. From a nutritional perspective, pea seed proteins are primarily composed of globulins and albumins, which largely determine their functional and nutritional properties [15,16]. These storage proteins are mainly represented by globulin fractions, including legumin (11S) and vicilin (7S), which differ in their structural, nutritional, and functional properties. Their relative proportions are particularly important for both nutritional quality and value for technological utilization, as they influence protein digestibility, amino acid balance, and functional characteristics such as gelation and emulsification. Pea proteins have proven to be valuable plant-based protein sources for both human and animal nutrition due to their favorable amino acid profile, good digestibility, and relatively low allergenicity [17,18].

Given the high degree of variability in the protein content of peas, attributable to the influence of environmental and management factors, the evaluation of pea performance should not be limited to grain quality alone. In addition to its nutritional value, pea plays an important role in cropping systems through biological nitrogen fixation. By reducing the need for synthetic nitrogen fertilizers, pea-based cropping systems may contribute to lowering environmental impacts and enhancing the sustainability of agricultural production systems [19,20]. Pea, particularly winter forms, is widely used as a forage crop and as a cover crop or green manure, where it contributes to soil fertility through biological nitrogen fixation [21]. Pea fixes atmospheric nitrogen through symbiosis with *Rhizobium leguminosarum bv. viciae*, contributing an estimated 50–150 kg N ha^−1^ to subsequent crops [22]. On average, pea fixes approximately 87 kg N ha^−1^ in aboveground biomass, with reported values ranging from 4 to 256 kg N ha^−1^ depending on environmental conditions and management practices [23]. Approximately 84% of the total aboveground nitrogen originates from atmospheric fixation, highlighting the strong contribution of pea to nitrogen cycling in agricultural soils. When incorporated at appropriate growth stages, legume-based green manures can further enhance the growth and nutrient availability for subsequent crops, improving overall system productivity [24,25]. As a preceding crop in rotation, pea has been shown to improve soil microbial communities and nitrogen balance while maintaining or increasing yields of following cereal crops under reduced input conditions [19]. In addition, pure stands of pea can produce approximately 2.6 t ha^−1^ of aboveground dry biomass [26].

Compared with the pure stand, the 2-species mixture yielded about 12% less biomass, while the multi-species mixtures (6- and 12-species) produced on average about 30% less. Under production conditions, the agronomic value of pea lies in its ability to fulfill multiple roles within a single cropping system or production region [27]. An integrated evaluation of pea performance across different agronomic use functions may therefore provide a more realistic and comprehensive understanding of its contribution to sustainable agriculture [6].

Despite extensive research addressing individual aspects of pea production—such as grain yield, protein quality, or use as a cover crop—these functions are often investigated separately and within narrowly defined experimental frameworks. Such fragmented approaches limit the understanding of how pea performs across multiple agronomic roles within diversified cropping systems. In particular, studies that jointly evaluate multiple agronomic functions under comparable environmental conditions remain limited. In temperate continental environments, where pea is frequently used for both production and ecological functions, studies that simultaneously consider grain yield, biomass production, and soil-related benefits remain relatively scarce.

The objective of this study was to evaluate the performance of a forage-type pea cultivar across multiple agronomic roles and to explore how different functional roles are expressed under varying management conditions. Rather than focusing on species-level comparisons, this study specifically examines the extent to which a single cultivar can express diverse agronomic functions under contrasting production scenarios. Given the limited number of genotypes included in Trial I and the differing treatment structures in Trials II and III, the results are not intended to represent population-level conclusions, but rather to illustrate functional patterns associated with this genotype under contrasting production scenarios.

In this context, the study explores whether different agronomic roles of pea are expressed as complementary (synergistic) or contrasting (trade-off) responses under varying management conditions, rather than assuming a uniform multifunctional outcome.

## 2. Results

### 2.1. Study Concept

This study presents an integrated interpretation of results obtained from a series of independent field trials evaluating multiple roles of winter pea (*Pisum sativum* L.). Although the experiments differed in design and specific objectives, their implementation under comparable environmental conditions and the use of a common variety, Kosmaj, enabled a comprehensive assessment of pea performance. Rather than emphasizing direct statistical comparisons among studies, the results have been arranged in accordance with the fundamental agronomic roles that were the subject of investigation. This structure allows for a clearer understanding of pea performance across different production contexts, including grain yield and protein accumulation, cover crop effects, biomass production, and green manure potential. The latter was reflected in soil nitrate nitrogen content and in the yield performance of the subsequent crop, in this case, maize (*Zea mays* L.). The following sections summarize the key findings of each experimental context, highlighting both the variability and consistency of pea performance across the evaluated functional roles.

### 2.2. Trial I—Grain Yield and Protein-Related Traits

**Figure 1:**
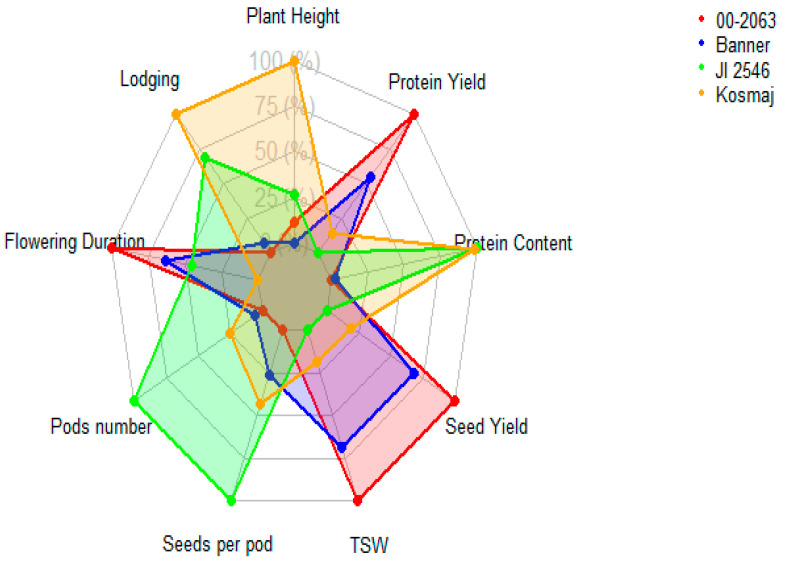
Radar chart showing phenotypic profiles of pea genotypes representing different functional types (forage, vegetable, dry-seed, and wild), based on morphological and yield-related traits. Values represent mean trait values across two growing seasons under temperate continental conditions at the experimental site. TSW—thousand-seed weight.

The radar chart (Figure 1) illustrates differences in phenotypic trait profiles among the analyzed pea genotypes representing different functional types, including a forage cultivar (Kosmaj), a vegetable-type line (00-2063), a dry-seed cultivar (Banner), and a wild Pisum accession (JI 2546), based on multiple morphological and yield-related traits (Supplementary S1). The corresponding numerical data is provided in the Supplementary Material. These differences are reflected in contrasting profiles of plant height, lodging tendency, seed yield, and thousand-seed weight (TSW) across genotypes. Moreover, patterns among traits could be observed from the radar plot: genotypes characterized by greater plant height and higher lodging tendency tended to have higher protein content but lower seed weight, whereas genotypes selected for grain production achieved higher seed yield and size at the expense of plant height. The forage-type variety Kosmaj exhibited the highest plant height (100.7 cm) among the genotypes, along with an increased lodging tendency (0.79), which distinguished it from both the vegetable-type (00-2063) and dry-type (Banner) genotypes. Kosmaj also showed relatively high protein content (28.8%), although seed yield and thousand-seed weight were lower compared to vegetable type (252.4 g) and dry-type genotypes (184.8 g). The vegetable-type 00-2063 displayed superior performance in seed yield (6244.3 kg ha^−1^) and thousand-seed weight (252.4 g), while maintaining moderate protein content. The dry-type variety Banner presented a balanced profile with intermediate values across most traits, particularly protein yield and flowering duration. The pea wild relative JI 2546 exhibited a unique profile, characterized by high pod number (12.7) and seeds per pod (5), but reduced seed size and overall grain yield, compared to the other genotypes.

**Figure 2:**
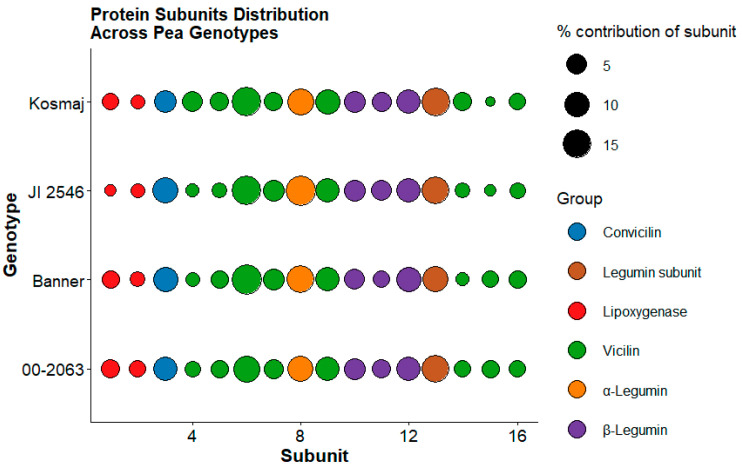
Bubble plot showing the relative abundance of major storage protein subunits in pea genotypes representing different functional types. Bubble size corresponds to the relative proportion (%) of each protein fraction. Values represent mean estimates across two growing seasons. Subunits include vicilin (7S), legumin (11S; α and β fractions), convicilin, and lipoxygenase-related fractions.

The bubble plot (Figure 2) revealed distinct differences in the relative contribution of major storage protein subunits among the four pea genotypes. Vicilin subunits represented the dominant protein fraction across all genotypes, particularly the 11.87–17.04% subunit range, although their relative proportions varied among genotypes. Kosmaj was characterized by a relatively high proportion of the main legumin subunit (14.30%) and substantial α-legumin content (12.33%), while maintaining considerable vicilin fractions. In contrast, JI 2546 exhibited the highest α-legumin proportion (17.51%) and elevated convicilin content, indicating a strong representation of globulin storage proteins. The vegetable- and dry-type genotypes (00-2063 and Banner) showed a more balanced arrangement between vicilin and legumin fractions, with consistent contributions of β-legumin subunits (ranging from approximately 4.38 to 10.01%). Lipoxygenase fractions were detected in all genotypes, but occurred in relatively low proportions. Overall, the results indicate genotype-specific protein composition patterns, with Kosmaj distinguished by a comparatively strong contribution of legumin subunits within an otherwise vicilin-dominated protein profile. These differences in protein subunit composition indicate genotype-specific variation in protein structure, which may affect functional properties and influence the suitability of different genotypes for specific food and feed applications.

### 2.3. Trial II—Pea in a Mixture as a Winter Cover Crop

**Figure 3:**
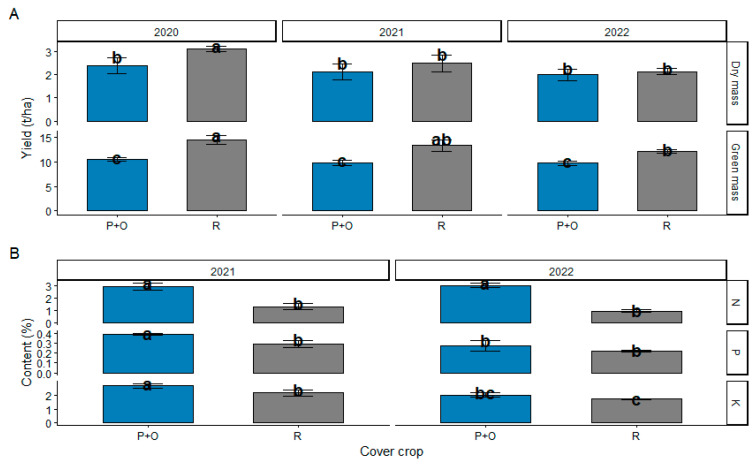
Biomass production (**A**) and nutrient content (**B**) of cover crops across years, under temperate continental conditions in Novi Sad, Serbia. Treatments include rye (*Secale cereale* L.; R) and a pea + oat (*Avena sativa* L.) mixture (P + O). Values are presented as mean ± standard deviation. Different letters indicate significant differences among treatments and years based on Tukey’s HSD test (*p* < 0.05). Nutrient content is expressed in % (dry matter basis).

Cover crop species differed in biomass production across years (Figure 3A). Rye (*Secale cereale* L.; R) consistently achieved higher biomass yields than the pea + oat (*Avena sativa* L.) mixture (P + O) in all three growing seasons. This difference was particularly evident in dry matter yield, which was used as the primary indicator for biomass comparison. Biomass production declined over time, with the highest values recorded in 2020 and the lowest in 2022. In 2022, dry biomass reached 2.12 and 1.99 t ha^−1^ for rye and P + O, respectively, with similar trends observed for green biomass. Overall, rye maintained a consistent yield advantage over the P + O mixture across all years, as supported by the differences indicated in Figure 3A. Nutrient concentrations were determined in 2021 and 2022 (Figure 3B). The P + O mixture showed higher nitrogen concentrations than rye, whereas differences in phosphorus and potassium were less pronounced. Potassium values ranged from 1.72–2.17% in rye and 2.02–2.67% in the mixture. Overall, rye exhibited superior biomass production, whereas the P + O mixture was characterized by higher nitrogen concentration, indicating a trade-off between biomass quantity and nitrogen enrichment.

**Figure 4:**
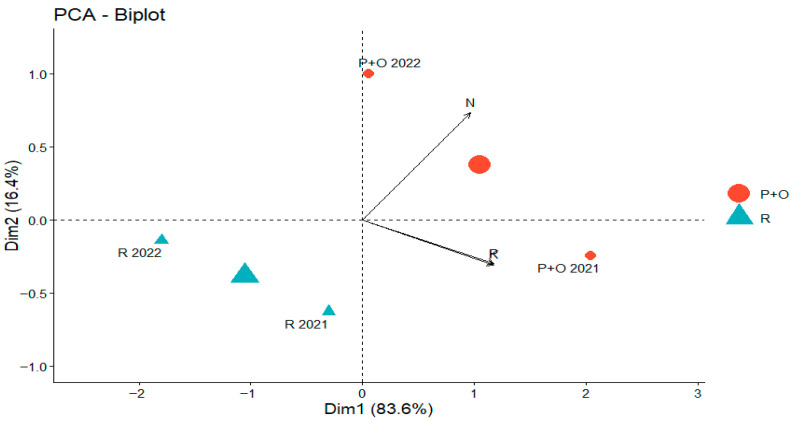
PCA biplot showing relationships between cover crop treatments (R, rye; P + O, pea–oat mixture) and measured variables across years. Dim1 (83.6%) separates treatments, while Dim2 (16.4%) reflects year-to-year variation.

To better understand the relationships among biomass and nutrient parameters, principal component analysis (PCA) was applied (Figure 4). The analysis revealed a clear separation between rye and the pea–oat mixture, reflecting differences in biomass production and nutrient composition. The pea–oat mixture was associated with higher nitrogen content in biomass prior to incorporation, whereas rye was more closely related to biomass production. Variation between years was also evident; however, it was primarily expressed as differences in the magnitude of response rather than changes in treatment ranking. This indicates that environmental conditions across years had a stronger influence on the observed variability than treatment effects. Overall, the results highlight a functional differentiation between treatments, where the pea–oat mixture is associated with improved nitrogen status, while rye is characterized by greater biomass production. The consistency of treatment grouping across years further supports the stability of these patterns under varying environmental conditions.

### 2.4. Trial III—Cover Crop Effects on Soil Fertility and Subsequent Maize Grain Yield

**Figure 5:**
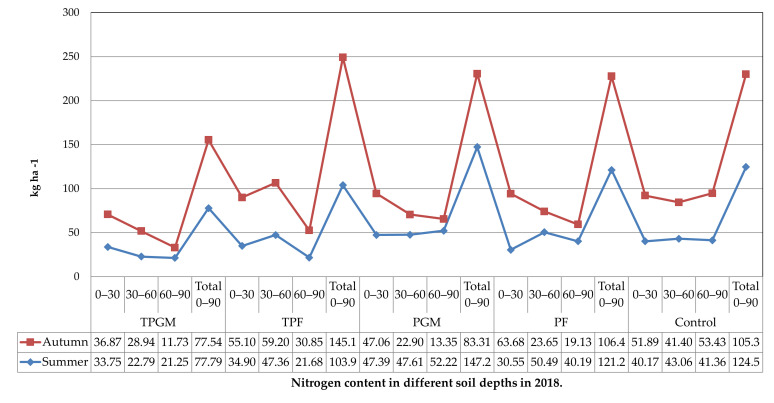
Soil nitrate nitrogen (N-NO_3_^−^) distribution across soil depths (0–30, 30–60, 60–90 cm) under different management treatments in 2018. Treatments include PTGM (pea + triticale green manure), PTF (pea + triticale forage), PGM (pea green manure), PF (pea forage), and Control (no cover crop). Values are expressed as kg ha^−1^.

**Figure 6:**
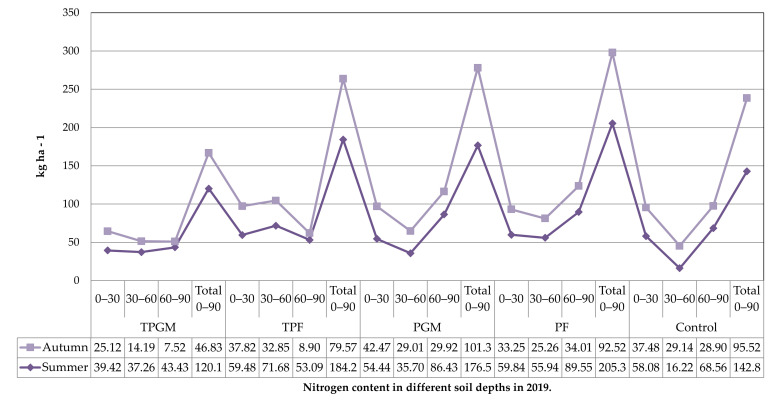
Soil nitrate nitrogen (N-NO_3_^−^) distribution across soil depths (0–30, 30–60, 60–90 cm) under different management treatments in 2019. Treatments include PTGM (pea + triticale green manure), PTF (pea + triticale forage), PGM (pea green manure), PF (pea forage), and Control (no cover crop). Values are expressed as kg ha^−1^.

The experiment included the following management treatments: PF (forage pea for forage), PGM (forage pea for green manure), PTF (forage pea + triticale (*Triticosecale*) for forage), PTGM (forage pea + triticale for green manure), and control (no cover crop). A three-factor ANOVA (Table 1) was conducted separately for each season to evaluate the effects of cover crop, utilization, and soil depth on N-NO_3_^−^ content. In summer 2018, a significant effect of cover crop was observed among treatments (F = 5.51, *p* = 0.020), whereas utilization and depth had no significant influence on N-NO_3_^−^ content. In autumn 2018, utilization and depth significantly affected N-NO_3_^−^ content, while cover crop showed no significant effect. During summer 2019, soil depth was the only significant factor, whereas cover crop and utilization were not statistically significant (Figure 5). In Autumn 2019, both cover crop and depth significantly influenced N-NO_3_^−^ content, while utilization remained non-significant (Figure 6). Overall, soil depth showed the most consistent effect across seasons, being significant in three out of four evaluations, whereas the effects of cover crop and utilization varied depending on the season (Table 1).

**Figure 7:**
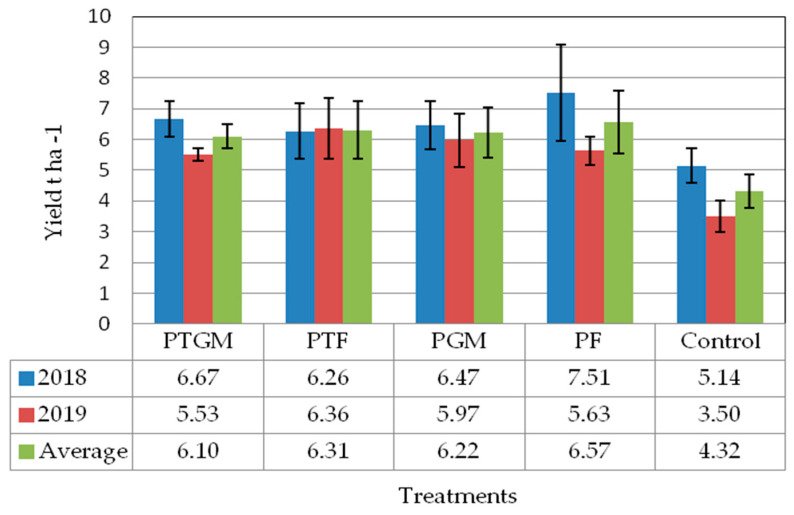
Maize grain yield following different cover crop treatments under temperate continental conditions. Treatments include PTGM (pea + triticale green manure), PTF (pea + triticale forage), PGM (pea green manure), PF (pea forage), and Control (no cover crop). Error bars represent standard deviation (SD) of three replicates.

Yield data were analyzed, and a two-way ANOVA (Table 2) was used to assess the effects of management treatment (cover crop × utilization) and year. The results indicated a significant effect of management treatment on yield (F = 6.70, *p* = 0.001), demonstrating that yield differed among the evaluated treatments. Year had a significant effect on yield, with overall differences observed between 2018 and 2019 (Figure 7). The model adequately described the data, with no significant lack-of-fit detected (Table 2).

**Figure 8:**
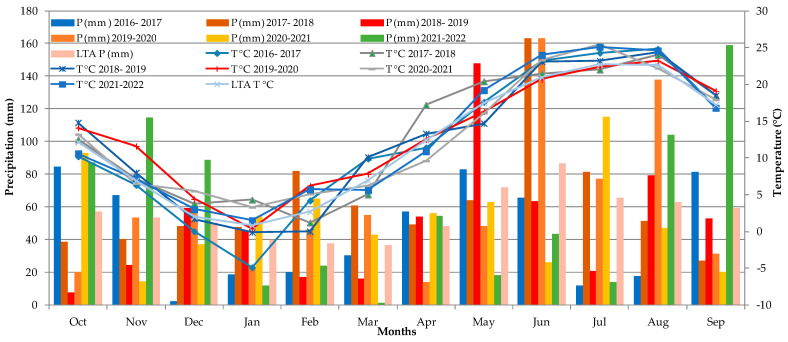
Long-term averages (LTA; 1964–2020), total monthly precipitation, and mean monthly air temperature for the hydrological years 2017–2018 to 2021–2022, recorded at the Rimski šančevi meteorological station located at the experimental site in Novi Sad, Serbia (distance: approximately 0.1 km). Precipitation is illustrated by bars associated with the left *y*-axis, while temperature is depicted by lines referenced to the right *y*-axis.

Meteorological conditions differed between the observed hydrological years of 2017–2018 and 2018–2019 (Figure 8), influencing soil nitrogen distribution and maize grain yield. The 2017–2018 season was characterized by more favorable precipitation distribution and slightly elevated temperatures during the growing period, compared to the long term average (Figure 8). In 2018, N-NO_3_^−^ decreased with depth across treatments, with the highest values observed in surface layers (Figure 5). Total nitrate content (0–90 cm) varied among treatments, with the highest values recorded under PGM. Maize grain yield in 2018 was generally higher, with PF achieving the maximum productivity (7.51 t ha^−1^), followed by PGM, while the control recorded the lowest grain yield (Figure 7). The 2018–2019 hydrological year showed less favorable precipitation patterns, including extremely low rainfall at the beginning of the season and increased variability thereafter. In 2019, N-NO_3_^−^ concentrations were substantially higher (Figure 6) compared with 2018 (Figure 5). In the summer sampling, the 0–30 cm layer ranged from 39.42 kg ha^−1^ (PTGM) to 59.84 kg ha^−1^ (PF). Unlike 2018, elevated N-NO_3_^−^ levels were also detected at 60–90 cm, particularly under PF (89.55 kg ha^−1^) and PGM (86.43 kg ha^−1^). Autumn sampling resulted in increased N-NO_3_^−^ values relative to summer, indicating continued mineralization. Despite higher total N-NO_3_^−^ (0–90 cm) in 2019, maize grain yield declined across most treatments. The PF treatment showed a notable yield reduction, whereas PTF recorded the highest yield in 2019 (6.36 t ha^−1^). Overall, interannual comparison revealed that improved precipitation distribution in 2017–2018 supported higher maize grain yields and surface N-NO_3_^−^ retention. In contrast, precipitation variability and elevated nitrogen redistribution in 2018–2019 were associated with reduced productivity.

## 3. Discussion

A distinctive aspect of the present study is its focus on a winter pea variety (Kosmaj) evaluated across multiple agronomic uses [28]. In contrast to conventional multi-genotype comparative studies, which primarily aim to identify superior genetic material, the cultivar-centered approach adopted here focuses on the functional plasticity of a single genotype across contrasting management contexts. This design reduces genetic variability as a confounding factor, allowing management and system-level effects to be interpreted with greater clarity, while not diminishing the importance of genetic diversity in practical agricultural systems. Such a perspective is particularly relevant under practical farming conditions, where decisions are typically made at the cultivar level. By assessing grain and protein traits, cover crop performance, and biomass or green manure potential within the same variety, the present study provides an integrated view of cultivar-level multifunctionality [6] under consistent environmental conditions, offering insight into how a single variety can be strategically positioned within diversified cropping systems. The agronomic profile observed for the forage-type pea evaluated in this study reflects its primary selection for vegetative biomass production rather than reproductive output, as evidenced by greater plant height and increased lodging tendency compared with grain-oriented genotypes. Such characteristics are typical of forage-type pea genotypes [2,6] and explain the lower expression of specific grain yield components, including seed yield and thousand-seed weight (Figure 1). Nevertheless, the relatively high protein content recorded (28.8%) indicates that forage-type genotypes may still contribute substantially to overall protein yield, particularly when protein concentration rather than grain yield alone is considered. This value is within or slightly above the typical range reported for pea grain protein content (approximately 20–25%) [8], further highlighting the potential of forage-type genotypes as a protein source. Similar patterns have been reported for forage-type pea, where enhanced vegetative growth and nitrogen accumulation are often associated with elevated seed protein levels [29,30]. In addition to differences in yield components, the contrasting phenotypic profiles observed among the analyzed genotypes illustrate the functional divergence between pea types selected for forage or grain production, as well as differences associated with distinct genetic backgrounds. Vegetable and dry-seed genotypes are typically selected for higher seed yield and larger seed size, traits directly associated with grain production and market value [1,12]. In contrast, forage-type pea is primarily bred for vigorous vegetative growth and biomass accumulation, which often results in taller plants and a greater susceptibility to lodging but may also support higher nitrogen accumulation and protein content in plant tissues [1,12]. Such differences in plant architecture and resource allocation reflect distinct breeding objectives and help explain the trait combinations observed in the present study. It should be noted that the relationships among traits were inferred from comparative phenotypic patterns rather than from formal correlation coefficients, and therefore represent indicative trends within the analyzed genotype set. Differences in protein subunit composition further emphasize functional variation among pea types, particularly between forage- and grain-oriented genotypes. Such variation in storage protein composition among pea genotypes has been widely documented and is known to influence both nutritional quality and technological functionality of pea proteins in food and feed applications [30,31]. In particular, the relative proportions of vicilin and legumin fractions affect protein digestibility, amino acid composition, and functional properties such as gelation and emulsification, which are important for both feed value and food processing. The higher contribution of legumin α subunits observed in this variety is consistent with previous studies linking these fractions to protein accumulation and techno-functional properties in pea seeds [7,32,33]. The predominance of legumin α subunits may have implications not only for total protein accumulation but also for processing quality, as legumin-rich fractions are often associated with improved gelation and emulsification properties in food applications [34]. The presence of lipoxygenase fractions may reflect metabolic activity related to protein synthesis and storage, as suggested in earlier reports [7,35]. When agronomic and protein-related traits are considered combined, forage-type pea may exhibit a combination of characteristics that extend beyond conventional grain production, aligning with recent European research emphasizing protein yield, functional quality, and resource-use efficiency in legume-based cropping systems [36]. This combination of vigorous vegetative growth and relatively high protein content also suggests a strong potential for biomass formation and nitrogen accumulation in plant tissues, linking grain-related traits with the ecological functions of pea observed in cover cropping and green manure systems.

Beyond grain and protein traits, the multifunctional role of pea becomes particularly evident when its biomass production and nutrient cycling functions are considered. Green biomass production of the selected winter cover crops was influenced by the type of cover crop, its biomass-forming capacity, and environmental conditions, particularly during the emergence phase, as reported in previous studies [37], although this aspect was not directly assessed in the present study. In the study by [38], compared with mixtures, a pure stand of forage pea produced greater aboveground biomass, whereas mixtures had an advantage in terms of belowground biomass and stability, indicating that the production potential of pea is primarily reflected in its aboveground biomass yield. A consistent pattern across the results was that pea–oat mixtures exhibited higher nitrogen concentrations, whereas rye-based systems produced greater total biomass, reflecting functional differences between legume-based and cereal-based cover crops. This functional differentiation is further supported by the PCA results (Figure 4), which clearly separate treatments according to their association with nitrogen content and biomass production. These differences should be interpreted in terms of contrasting functional roles rather than as a direct comparison between species. Rye, as a cereal cover crop, is typically associated with high biomass production, whereas legume-based mixtures such as pea–oat contribute to enhanced nitrogen concentration and improved residue quality. Temporal variation in dry biomass production observed across years underscores the strong influence of climatic conditions on cover crop performance and reinforces the need to interpret biomass-related results within a multi-year framework. Similar observations have been reported by [39] who showed that forage pea biomass production strongly depends on agroecological conditions, while mixtures with cereals such as triticale tend to produce higher overall biomass than pure pea stands. When pea is grown in an intercropping system with other species, it significantly contributes to nitrogen accumulation in soil. In the present study, the P + O mixture consistently exhibited higher nitrogen concentrations (2.93–3.01%) compared with R grown as a sole crop (0.93–1.29%) in both 2021 and 2022. Although R produced greater total biomass across all three years, the elevated N concentration in the mixture indicates a higher potential for nitrogen input to subsequent crops. These findings are consistent with previous studies reporting that oat grown in combination with legumes demonstrate high and stable biomass production and enhanced N accumulation [40]. While phosphorus and potassium concentrations showed comparatively smaller differences between treatments, their variation appeared more closely related to biomass production and species-specific nutrient uptake rather than biological nitrogen fixation. In the present study, phosphorus concentrations showed only minor differences between treatments, whereas potassium concentrations tended to be higher in the pea–oat mixture compared with rye grown as a sole crop. Cereal cover crops such as rye, oat, and barley are often associated with high biomass production; however, winter legumes such as pea can also produce substantial biomass, particularly under favorable conditions or when grown in mixtures [29,40]. Higher biomass production in cereals generally results in greater total nutrient uptake, particularly potassium, although their tissue nitrogen concentration is typically lower [41]. Legumes, by contrast, contain higher nitrogen concentrations and greater N content per unit biomass due to biological N fixation and lower C:N ratios. Consequently, their residues tend to decompose more rapidly and contribute more plant-available nitrogen to the soil [42,43,44,45].

In Trial III the results indicate that management treatments strongly influenced soil nitrate nitrogen (N-NO_3_^−^) distribution and maize grain yield, with responses varying between hydrological years due to contrasting meteorological conditions (Figure 8). The observed variability between years indicates a strong influence of environmental conditions, particularly precipitation and temperature patterns, on system performance. However, the generally consistent differences among treatments across years suggest a stable response under contrasting environmental conditions, highlighting the robustness of the system. At the same time, consistent differences among treatments across years suggest that management effects also contributed to the observed responses, although their magnitude depended on seasonal conditions. Maize production in Vojvodina province is predominantly conducted under rainfed conditions [46]. In 2018, characterized by more favorable precipitation distribution (Figure 7), precipitation during the cover crop growing period likely enhanced biomass production and nitrogen accumulation, which in turn improved soil nutrient availability for the subsequent maize crop. Under these conditions, treatments including forage pea (PGM, PF, PTF, PTGM) promoted higher nitrate concentrations in the surface soil (0–30 cm), while N-NO_3_^−^ decreased with depth, as shown in Figure 5 and Figure 6. The PGM treatment produced the highest total N-NO_3_^−^, reflecting substantial nitrogen release from incorporated legume biomass. Nitrate availability in the top soil layer contributed to improved maize performance, as evidenced by the highest yield under PF. These findings are comparable with those demonstrating that legume residues increase mineral nitrogen availability and improve nitrogen uptake efficiency in subsequent cereal crops [25,47,48]. In contrast, the 2019 season experienced uneven precipitation (Figure 8) and early drought, which altered nitrogen dynamics. Although total N-NO_3_^−^ levels were higher than in 2018, elevated nitrate concentrations were observed in deeper layers (60–90 cm), particularly under PF and PGM, suggesting increased nitrate leaching as a result of excessive rainfall in July and August or reduced crop uptake. Despite greater nitrate availability, maize grain yields declined across most treatments, indicating that high temperatures in August and uneven rainfall patterns have restricted nitrogen utilization. Comparable discrepancies between soil nitrate abundance and crop performance have been frequently documented under various abiotic stress conditions [49]. Nitrogen availability alone does not necessarily lead to improved crop productivity [50], since nitrogen uptake and use are strongly conditioned by water availability and environmental factors; moreover, the same authors [50] reports that excessive nitrogen supply is associated with reduced grain yield under both water-limited and well-watered conditions, alongside an increased risk of adverse nitrogen losses. Among treatments, PTF achieved the highest maize grain yield in 2019, suggesting that mixed forage systems may enhance resilience under unfavorable moisture regimes, potentially through improved soil structure [51,52]. Cover crops generally reduce soil evaporation and evapotranspiration while improving soil water storage, although their effects on subsequent crop yield and water use efficiency are highly dependent on soil, climate, and residue management. Maintaining adequate biomass, retaining residues, and ensuring a minimum three-week interval between cover crop termination and planting of the succeeding crop are critical for optimizing soil water recharge, yield stability, and water use efficiency [53]. Across both years, the control consistently showed lower nitrate levels and maize grain yields, highlighting the agronomic benefits of integrating forage pea (Figure 7). As a legume, forage pea contributes to biological nitrogen fixation, enhances soil nitrogen pools, and supports more sustainable nutrient cycling [21,54]. The incorporation or utilization of pea biomass represents an effective strategy for reducing dependency on synthetic nitrogen fertilizers while maintaining crop productivity [6,55,56,57]. Overall, the study indicates that pea-based systems significantly modify soil nitrate dynamics and influence maize grain yield, and this finding is consistent with previous research [26,58,59]; however, their effectiveness depends on climatic conditions, particularly precipitation patterns. It should be noted that biomass production was not directly quantified in Trial III, and therefore interpretations regarding nitrogen inputs are based on observed soil responses and supported by previous research [39]. Aligning legume management with climatological variability remains essential for optimizing nitrogen use efficiency and yield stability [55]. Optimal nitrogen management—aligned with water availability and crop requirements is essential for achieving stable yields and reducing the risk of nitrogen losses [50,60].

From a practical perspective, the results suggest that the role of pea within cropping systems should be aligned with specific production goals. When biomass production and soil nitrogen inputs are prioritized, pea-based mixtures or green manure systems may be most suitable. In contrast, when grain production and protein yield are the primary objectives, management practices should focus on optimizing reproductive performance. Under variable climatic conditions, integrating pea into mixed or rotational systems may enhance system resilience and contribute to more stable production outcomes. The overall results from the three studies provide complementary evidence of the multifunctional potential of pea, reflected in its contributions to grain and protein production, biomass yield, and intercropping systems, particularly with species from the Poaceae, as well as green manuring and soil nitrogen dynamics. It should be noted that the present study is variety-focused, and therefore the results should not be generalized to the entire pea species without further validation across a broader range of genetic material. Although these functions were not assessed simultaneously within a single experiment, the combined findings offer a broader perspective on the role of pea in diversified cropping systems under the same environmental conditions. The series-of-trials approach emphasizes that the role of pea in sustainable agriculture cannot be reduced to a single function or parameter, but rather lies in its versatility and adaptability within diversified cropping systems. In this context, the results obtained across the three trials illustrate how a single pea cultivar can simultaneously contribute to grain and protein production, biomass generation, and soil nitrogen dynamics. Such cultivar-level multifunctionality highlights the strategic role of pea in integrated and resource-efficient cropping systems.

## 4. Materials and Methods

### 4.1. Experimental Site

**Scheme 1:**
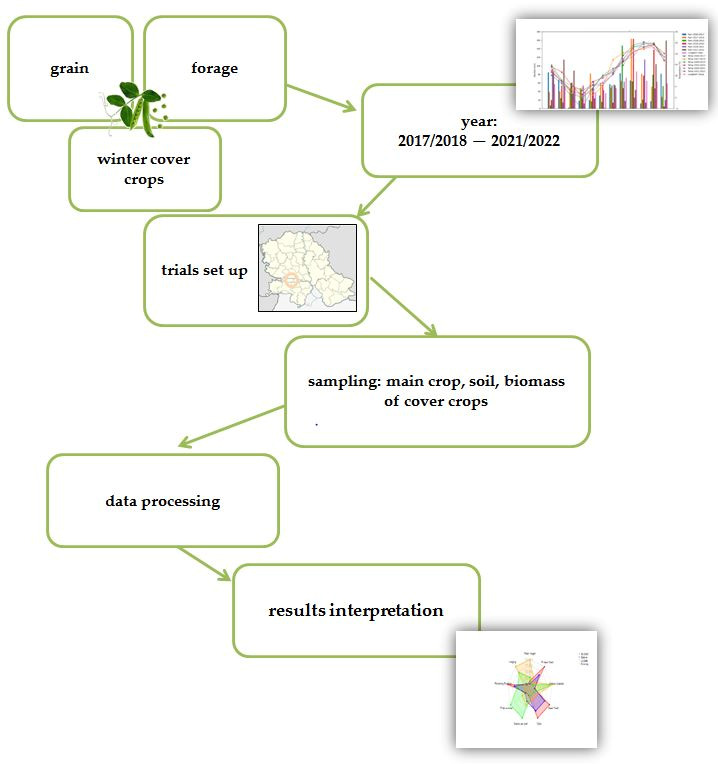
Experimental site, trial structure, and dynamics of the experiment conducted at Rimski šančevi experimental station, Novi Sad, Serbia, during the period 2017–2022.

All field experiments (Scheme 1) were conducted at the Rimski šančevi experimental station of the Institute of Field and Vegetable Crops, Novi Sad, Serbia (45°19′ N, 19°50′ E; approximately 80 m a.s.l.). The site is characterized by a temperate continental climate with pronounced seasonal variation in temperature and precipitation. According to long-term meteorological records, the average annual air temperature is approximately 11.4 °C, while the average annual precipitation is about 640 mm. The soil at the experimental site is classified as Chernozem according to the World Reference Base for Soil Resources (WRB) classification [61], and is characterized by favorable physical and chemical properties for pea cultivation.

### 4.2. Weather Conditions During the Research Period

Monthly air temperature and precipitation data were obtained from the meteorological station Rimski šančevi, located approximately 0.1 km from the experimental site, covering five hydrological years (2017–2018 to 2021–2022). Long-term average (LTA) values were calculated based on the reference period 1964–2020 using data from the Republic Hydrometeorological Service of Serbia (https://www.hidmet.gov.rs/, accessed on 15 October 2025).

Meteorological conditions exhibited pronounced interannual variability, with deviations from long-term averages observed for both precipitation and air temperature. The 2017–2018 season was characterized by moderate autumn and winter precipitation followed by increased rainfall in late spring, while temperatures were generally above average. In contrast, the 2018–2019 season showed reduced precipitation during autumn and winter, followed by higher rainfall in spring. Subsequent years also showed variable precipitation distribution and elevated temperatures during the growing season (Figure 8).

In this multifunctional study, the forage pea (*Pisum sativum* L.) variety *Kosmaj*, which is frost-tolerant (surviving temperatures down to −17 °C) and suitable for grain, biomass, hay, silage, and haylage production, was used.

Although the experiments included in this study differ in their specific objectives and designs, they collectively address key functional aspects of pea performance under diverse agronomic conditions. The aim of this study was therefore to provide a complementary, cultivar-level perspective by synthesizing results from multiple experiments, rather than to perform a direct comparison across treatments or systems.

The three experimental designs and procedures involving this variety are described below.

### 4.3. Trial I—Grain Yield and Protein of Forage Pea-Related Traits

Trial I evaluated morphological and agronomic variation in pea, related to grain yield and seed protein traits, over a two-year period (2019–2020). The experiment was originally established within a broader breeding-oriented research program [62] using an augmented block design with partial replications, allowing efficient evaluation of a large number of genotypes under field conditions. The experiment was conducted in the same field across years; however, treatments were re-established each season on newly assigned plots within the field and were not repeatedly applied to the same plot positions. Plot layout was re-randomized annually to minimize potential residual effects from previous treatments. All plots were managed according to standard agronomic practices. The size of the individual plot was 1 × 5 m (5 m^2^). For the present study, four genotypes representing contrasting functional types were selected: the forage pea variety (Kosmaj), a protein-type variety (Banner), a vegetable-type research line (00-2063), and a wild-type Pisum accession (JI 2546). These genotypes enabled a comparative assessment of yield and protein-related traits across different usage types. Morphological and agronomic traits were evaluated following standard descriptors for pea. The assessed traits included plant height, lodging score, flowering duration, seed yield (kg ha^−1^), seed protein content (%), protein yield (kg ha^−1^), and selected yield components such as number of pods per plant, number of seeds per pod, and thousand seed weight (g). Yield components were determined from 10 representative plants per plot. Thousand-seed weight (TSW) was determined according to standard ISTA procedures [63]. Seed protein content was determined on dry whole seed samples of 150 g, using FT-NIRS (Antaris II, Thermo Scientific, Waltham, MA, USA) with OMNIC TM (Series Software 9), based on calibration models developed at the Institute of Field and Vegetable Crops, Novi Sad. Samples were dried to constant weight prior to analysis. Seed yield was determined on a plot basis and adjusted to standard moisture content [63].

### 4.4. Trial II—Pea as a Winter Cover Crop

Trial II assessed the performance of forage pea as an intercropping cover crop over a three-year period (2019–2022). The experiments were conducted following wheat as the preceding crop. The total area under trial was 30 m × 90 m. Individual plot size per cover crop was 30 m × 30 m per winter cover crop. The experiment was conducted using a randomized block design with 4 replicates per treatment. Each replicate consisted of an independent plot, and all measurements were performed at the plot level. Utilization was defined as a management factor representing the intended use of cover crop biomass as green manure (biomass incorporation into the soil). In the trials, pea (P) was sown as a winter cover crop in open-field production systems within a sowing window extending from October to April/May (Table 3 and Table 4). Pea was established in mixed sowing with oat (O) (*Avena sativa* L.) to optimize biomass production and reduce interspecific competition, whereas rye (R) (*Secale cereale* L.) was grown as a sole (pure) cover crop to determine the specific effect of pea grown in association with a cereal species. The methodology focused on monitoring cover crop growth and biomass accumulation. Before mulching in the cover crop and disking in April/May (Table 3 and Table 4), biomass samples were taken for further analysis. Four samples per cover crop (1 m^2^) were taken on the day before winter cover crop mechanical termination, followed by biomass incorporation into soil. Measurements included biomass yield and quality parameters relevant for green manure utilization.

### 4.5. Trial III—Cover Crop Effects on Nitrate N Content and Subsequent Maize Grain Yield

Trial III evaluated the effects of winter cover crops on nitrate nitrogen content (N-NO_3_^−^) and the grain yield of subsequent maize (*Zea mays* L.) during the 2017–2019 period. The experiment was arranged in a randomized complete block design with three replications. Winter cover crop treatments consisted of: (I) a single-species forage pea (P) (*Pisum sativum* ssp. *arvense*), and (II) a mixture of forage pea and triticale (PT) (×*Triticosecale*). Each cover crop treatment was divided into the following two sub-plots (way of using cover crops): incorporation into the soil as green manure (GM) and mowing with biomass removal (forage) followed by ploughing (F). Accordingly, five treatments were established: PTGM, PTF, PGM, PF, and a control without cover crops (Control). Individual plot size was 6 m × 4 m (24 m^2^). Seed rates of cover crops are summarized in Table 3. Following ploughing and seedbed preparation, maize was sown in early June, with a spacing of 23 cm between plants within rows and 70 cm between rows, corresponding to a planting density of 62,000 plants ha^−1^. The maize grain yield was determined at harvest time (October) by randomly selecting 15 plants per plot across three replicates. The grain yield values were standardized to a moisture content of 14%. Soil samples for nitrate nitrogen (N-NO_3_^−^; Nmin) determination were collected from three depths (0–30, 30–60, and 60–90 cm) at two sampling times: 3–4 weeks after cover crop incorporation (prior to maize sowing in June) and after maize harvest. All relevant information on the timing of specific cultivation practices is presented in Table 4.

The detailed information on sowing rates, as well as sowing and harvesting dates for all three trials, is presented in Table 3 and Table 4.

**Table 3:** Sowing rates and crop composition in Trials I–III.

Trial	Seed Amount	Crop
Trial I	120 kg ha^−1^	Pea
Trial II	210 kg ha^−1^	Rye
	140 kg ha^−1^	Pea
	30 kg ha^−1^	Oat
Trial III	120 kg ha^−1^	Forage pea
	90 + 30 kg ha^−1^	Forage pea + Triticale

**Table 4:** Field operations and dates across Trials I–III.

Trial	Year	Crop	Operation	Date
Trial I	2019	Pea	Sowing	3 March
			Harvest	10 July
	2020	Pea	Sowing	1 March
			Harvest	21 July
Trial II	2019–2020	Rye Pea + Oat	Sowing Incorporation	27 October 24 April
	2020–2021	Rye Pea + Oat	Sowing Incorporation	15 October 5 May
	2021–2022	Rye Pea + Oat	Sowing Incorporation	20 October 11 May
Trial III	2017–2018	Forage pea Forage pea + triticale	Sowing	8 November
			Sowing	8 November
			Incorporation	18 May
		Maize (subsequent crop)	Sowing	15 June
			Soil sampling	1 July
			Harvest	18 October
			Soil sampling	20 October
	2018–2019	Forage pea Forage pea + triticale	Sowing	7 November
			Sowing	7 November
			Incorporation	22 May
		Maize (subsequent crop)	Sowing	11 June
			Soil sampling	25 June
			Harvest	23 October
			Soil sampling	25 October

### 4.6. Measurements and Analytical Determination

Average soil chemical properties across the experimental years for trials under trials I, II, and III are presented in Table 5. In autumn, disturbed field-moist soil samples (1 kg per plot) were collected from the top 0–30 cm of the soil profile. Soil pH was determined in the suspension of soil in KCl and H_2_O, by pH meter (MA 3657, METREL, Horjul, Slovenia). Calcium carbonate (CaCO_3_) content was determined volumetrically using a Scheibler calcimeter (Gabbrielli, Mie, Japan). Humus content was determined by oxidizing the soil organic matter using potassium dichromate [64]. Mineral nitrogen in the soil was extracted with 2 M KCl at a 1:4 soil-to-solution ratio (weight basis) and quantified using steam distillation [65]. Available phosphorus and potassium were determined using the ammonium lactate method. Phosphorus was measured by spectrophotometry and potassium by flame photometry [66]. Mineral nitrogen forms (NO_3_–N) in the soil layers 0–30, 30–60 and 60–90 were analyzed following the method of Wehrmann and Scharpf [67].

### 4.7. Statistical Analysis

Statistical analyses were performed using the R software environment (version 4.2.2; R Core Team, Vienna, Austria [68]). The analytical approach was adapted to the specific experimental design and objectives of each trial, while maintaining a consistent statistical framework across the study. Prior to analysis, data were inspected for outliers and checked for assumptions of normality and homogeneity of variance where applicable. Descriptive statistics were used to summarize the main characteristics of the datasets. Analysis of variance (ANOVA) was applied to assess the effects of experimental factors and their interactions. Post hoc tests were applied where appropriate. For exploratory and comparative purposes, multivariate and graphical visualization techniques were employed. A radar plot was constructed using the fmsb package [69] to visualize comparative morphological and agronomic trait profiles among genotypes, while bubble plots, boxplots, and other visualizations were generated using the tidyverse [70] and ggplot2 [71] packages. Bar charts with error bars were generated using XLSTAT [72].

Statistical models were specified separately for each trial according to the experimental design. In Trial I, due to the augmented design without full replication, results were analyzed descriptively, and no formal inferential model was applied. In Trial II, biomass and nutrient data were summarized as mean ± standard deviation, and differences between treatments within each year were assessed using Tukey’s HSD test. Principal component analysis (PCA) was additionally applied to explore relationships among biomass production and nutrient composition across years. In Trial III, a factorial ANOVA model was applied, including cover crop treatment, utilization (green manure vs. forage), and soil depth as fixed effects, along with their interactions. Year was treated as a fixed factor because the study aimed to compare treatment responses under the specific environmental conditions of the experimental years, rather than to generalize across a random sample of years. Analyses were conducted separately for each sampling season (summer and autumn) due to differences in sampling timing and system conditions. The year was treated as a fixed factor, as the study focused on specific environmental conditions during the experimental period rather than inference across a broader population of years. A combined analysis across seasons was not performed, as the data represent distinct system states (pre- and post-maize), which would violate the assumptions required for a unified repeated-measures model. Replication was included as a random effect. All models were evaluated with appropriate interaction terms, and significance was assessed at standard probability levels.

## 5. Conclusions

The findings presented in this study are based on a series of complementary trials in which different functional aspects were evaluated separately. Therefore, the results should be interpreted within this experimental framework and not as evidence of simultaneous multifunctionality within a single system. This study suggests that forage-type pea has the potential to contribute to multifunctional roles within sustainable agricultural production systems. As a leguminous crop, pea can combine grain production, protein accumulation, and biomass formation, thereby contributing to plant-based protein supply and supporting diversified cropping systems. The results from Trial I highlight differences among pea types in terms of agronomic and protein-related traits, while Trial II and III demonstrate the role of pea as cover crop and pea–cereal systems with respect to biomass production and soil nitrogen dynamics. In these systems, pea contributes to nutrient cycling and may reduce reliance on mineral nitrogen fertilizers, while also supporting the performance of subsequent crops in rotation. Pea–cereal mixtures showed potential for complementary resource use, contributing to biomass production and supporting forage and green manure functions. However, these effects should be interpreted within the context of species-specific growth characteristics and management conditions. Overall, the results suggest that multifunctional pea genotypes represent a valuable option in sustainable cropping systems, but their use should be adapted to specific production goals and environmental conditions.

## Figures and Tables

**Table 1:** Analysis of variance (ANOVA) of soil nitrate nitrogen (N-NO_3_^−^) content.

		Summer 2018		Autumn 2018		Summer 2019		Autumn 2019	
Source	DF	F-Value	*p*-Value	F-Value	*p*-Value	F-Value	*p*-Value	F-Value	*p*-Value
Cover Crop	2	5.51	0.020	0.57	0.579	1.74	0.218	5.32	0.022
Utilization	1	0.00	0.996	5.68	0.034	1.92	0.191	0.69	0.421
Depth	2	0.95	0.413	18.98	0.000	5.22	0.023	5.52	0.020
Error	12								
Total	17								

**Table 2:** Analysis of variance (ANOVA) for maize grain yield.

Source	df	MS	F	*p*
Management treatment	4	4.8907	6.70	0.001 **
Year	1	7.6934	10.53	0.003 **
Residuals	24	0.47303		

Significance levels: ** *p* < 0.01.

**Table 5:** The average soil chemical properties of the test plots over the course of the experiments.

Trial	Year	pH KCl	pH H_2_O	CaCO_3_	Humus (%)	N (%)	(mg kg^−1^ Soil)	
							P_2_O_5_	K_2_O
**Trial I**	2019	7.40	8.05	6.33	2.23	0.16	25.20	22.00
	2020	7.42	8.10	6.89	2.34	0.17	24.78	23.50
**Trial II**	2019	7.45	8.17	5.87	2.30	0.17	17.91	20.90
	2020	7.20	8.21	4.41	2.24	0.16	16.19	20.23
	2021	7.43	8.41	7.77	2.20	0.15	16.93	19.30
**Trial III**	2017	7.48	8.14	8.96	2.59	0.19	17.13	23.67
	2018	7.43	8.11	8.68	2.53	0.19	18.41	23.43
	2019	7.23	8.12	9.48	2.69	0.20	14.54	23.21

## Data Availability

The original contributions presented in this study are included in the article/Supplementary Materials. Further inquiries can be directed to the corresponding author.

## Supplementary Materials

The following supporting information can be downloaded at: https://www.mdpi.com/article/10.3390/plants15081226/s1, S1: Mean values of morphological and agronomic traits used for radar plot construction, averaged across two years for all analyzed pea genotypes. S2: Raw biomass yield data (t ha^−1^) of rye (R) and pea + oat mixture (P + O) across treatments, replicates, and years.
