# Supplementary material for: Assessing Multiple Agronomic Functions of a Winter Pea (Pisum sativum L.) Variety Across Different Uses
Source: Plants (Basel). 2026 Apr 16;15(8):1226. doi: 10.3390/plants15081226 (PMC13120300; doi:10.3390/plants15081226)
Supplement: Supplementary file 1 [file plants-15-01226-s001.zip › plants-4234698-supplementary-S2.pdf]

Supplement S2. Raw biomass yield data (t $ha^{-1}$) of rye (R) and pea + oat mixture (P+O) across treatments, replicates, and years

**Biomass yield in t $ha^{-1}$**

| Cover crop | repeat | year | Dry matter (**t $ha^{-1}$**) |
|---|---|---|---|
| R | 1 | 2020 | 3.05 |
| R | 2 | 2020 | 3.19 |
| R | 3 | 2020 | 3.24 |
| R | 4 | 2020 | 2.99 |
| P+O | 1 | 2020 | 2.1 |
| P+O | 2 | 2020 | 2.35 |
| P+O | 3 | 2020 | 2.89 |
| P+O | 4 | 2020 | 2.22 |
| R | 1 | 2020 | 3.93 |
| R | 2 | 2020 | 3.87 |
| R | 3 | 2020 | 3.75 |
| R | 4 | 2020 | 3.85 |
| P+O | 1 | 2020 | 2.39 |
| P+O | 2 | 2020 | 2.65 |
| P+O | 3 | 2020 | 2.26 |
| P+O | 4 | 2020 | 2.37 |
| R | 1 | 2021 | 2.98 |
| R | 2 | 2021 | 2.31 |
| R | 3 | 2021 | 2.52 |
| R | 4 | 2021 | 2.16 |
| P+O | 1 | 2021 | 1.82 |
| P+O | 2 | 2021 | 1.96 |
| P+O | 3 | 2021 | 2.04 |
| P+O | 4 | 2021 | 2.61 |
| R | 1 | 2021 | 3.97 |
| R | 2 | 2021 | 3.92 |
| R | 3 | 2021 | 4.05 |
| R | 4 | 2021 | 3.72 |
| P+O | 1 | 2021 | 3.95 |
| P+O | 2 | 2021 | 4.28 |
| P+O | 3 | 2021 | 4.31 |
| P+O | 4 | 2021 | 4.27 |
| R | 1 | 2022 | 2.3 |
| R | 2 | 2022 | 2.1 |
| R | 3 | 2022 | 2 |

| | | | |
|---|---|---|---|
| R | 4 | 2022 | 2.1 |
| P+O | 1 | 2022 | 1.85 |
| P+O | 2 | 2022 | 1.7 |
| P+O | 3 | 2022 | 2.2 |
| P+O | 4 | 2022 | 2.2 |
| R | 1 | 2022 | 1.85 |
| R | 2 | 2022 | 1.6 |
| R | 3 | 2022 | 1.95 |
| R | 4 | 2022 | 2.1 |
| P+O | 1 | 2022 | 2.35 |
| P+O | 2 | 2022 | 2.6 |
| P+O | 3 | 2022 | 2.7 |
| P+O | 4 | 2022 | 2.4 |
